# Involvement of Alternative Splicing in Barley Seed Germination

**DOI:** 10.1371/journal.pone.0152824

**Published:** 2016-03-31

**Authors:** Qisen Zhang, Xiaoqi Zhang, Songbo Wang, Cong Tan, Gaofeng Zhou, Chengdao Li

**Affiliations:** 1 Australian Export Grains Innovation Centre, 3 Baron-Hay Court, South Perth, WA 6155, Australia; 2 Western Barley Genetics Alliance, Murdoch University, 90 South Street, Murdoch, WA 6150, Australia; 3 BGI-Shenzhen, Beishan Industrial Zone, Yantian District, Shenzhen, 518083, China; 4 Department of Agriculture and Food Western Australia, 3 Baron-Hay Court, South Perth, WA 6155, Australia; International Centre for Genetic Engineering and Biotechnology, ITALY

## Abstract

Seed germination activates many new biological processes including DNA, membrane and mitochondrial repairs and requires active protein synthesis and sufficient energy supply. Alternative splicing (AS) regulates many cellular processes including cell differentiation and environmental adaptations. However, limited information is available on the regulation of seed germination at post-transcriptional levels. We have conducted RNA-sequencing experiments to dissect AS events in barley seed germination. We identified between 552 and 669 common AS transcripts in germinating barley embryos from four barley varieties (*Hordeum vulgare* L. Bass, Baudin, Harrington and Stirling). Alternative 3’ splicing (34%-45%), intron retention (32%-34%) and alternative 5’ splicing (16%-21%) were three major AS events in germinating embryos. The AS transcripts were predominantly mapped onto ribosome, RNA transport machineries, spliceosome, plant hormone signal transduction, glycolysis, sugar and carbon metabolism pathways. Transcripts of these genes were also very abundant in the early stage of seed germination. Correlation analysis of gene expression showed that AS hormone responsive transcripts could also be co-expressed with genes responsible for protein biosynthesis and sugar metabolisms. Our RNA-sequencing data revealed that AS could play important roles in barley seed germination.

## Introduction

Seed germination initiates a transition of biological activities from quiescent to active statuses. Most of the machineries for energy metabolism and protein synthesis are present in dehydrated seeds, but integrity and physiochemical status are different from living cells. One of biological activities quickly activated after seed imbibition is the resumption of respiration. An increase in gas exchanges was observed within minutes upon seed imbibition in maize embryos [1]. Membrane repair is another important biological activity observed in cotton cotyledons [2]. Membrane phospholipid is in a gel status in dry seed [3]. Influx of water onto cells during imbibition disturbs the membrane structure. Solute leakages from cells were observed [4]. Mitochondrial repair is important in the initiation of seed germinations, as shown by the increased in respiration and biosynthesis of cytochrome c oxidase [5]. Cell wall modification was also very active in the germinating embryos [6]. However, limited information is available on whether these processes involved alternatively spliced transcripts and which AS genes played important roles in germinating embryos.

Alternative splicing (AS) increases transcriptomic plasticity and proteomic diversity, which extends gene-coding capacities in eukaryotic genomes. It regulates cell differentiation and many developmental processes [7,8,9]. It generates more than one mRNA variant from precursor mRNA transcripts and regulates transcript levels by introducing premature termination [10,11]. It produces transcript isoforms with altered sequences which target different cellular locations or have different functionalities [12]. It plays important roles in co- and post-transcriptional regulations in *Arabidopsis* [7,8,9] and in soybean embryos [13]. It is involved in many biological processes including nutrient uptake [14], circadian clock, organ differentiation, biotic and abiotic stress responses in *Arabidopsis*; oil formation in soybean; and temperature and light responses in *Physcomitrella patens* [7,8,9,15,16].

Barley is an important cereal crop ranking fourth in worldwide cereal production after maize, rice and wheat. It has been used as animal feed and for health food ingredients, but good quality barley is mainly used for malt in the brewing industry, which is a controlled seed germinating process. Thus, understanding of seed germination will provide valuable information for controlling malting and brewing qualities and also for understanding of seed germination properties such as germination rates and seed vigour. We conducted RNA sequencing (RNA-seq) experiments to dissect common AS events and transcriptional activities in germinating embryos of four barley varieties. Between 552 and 669 common AS transcripts were identified. The AS transcripts were predominantly mapped to pathways/ complexes of ribosome, RNA transporting, spliceosome, oxidative phosphorylation, mRNA surveillance, carbon metabolism and plant hormone signal transduction pathways. Furthermore, since AS transcripts were predominantly found not only in protein and energy metabolic pathways, but also in regulatory pathways including spliceosome and hormone signal transduction, they could play very important roles in seed germination.

## Materials and Methods

### Plant materials

Four barley varieties of *Hordeum vulgare* L Bass, Baudin, Harrington and Stirling were selected for this study, since we wanted to dissect common AS events. Seeds of the four varieties were obtained from Department of Agriculture and Food, Western Australia. These seeds were harvested from the same field trial in Western Australia. The seeds were tested without pre-harvest sprouting damages and dormancy before they were used for germination to extract RNA. For RNA preparation, about 400 seeds were sterilized with 1% (w/v) hypochlorite solution for 15 min, rinsed with running water and transfered them to 10 cm Petri dishes with two layers of filter paper and 16 ml distilled water each. After imbibing for 24 h or 48 at 21 ^o^C in dark, germinating embryos were separated from endosperms and placed in a microfuge tube on dry ice. The embryos were stored at –80 ^o^C before RNA extraction. Total RNA was extracted with phenol-SDS reagents (http://onlinelibrary.wiley.com/doi/10.1002/0471142727.mb0403s09/pdf).

### Sequencing and bioinformatics analysis

Details of RNA sequencing and bioinformatics analysis methodologies were published previously [6]. A brief summary was described below.

#### RNA-seq library preparation and sequencing

Total RNA was extracted and treated with DNase to remove genomic DNA contamination. The quality of the RNA extract was assessed using the Agilent 2100 Bioanalyzer system with a minimum RNA integrated number value of 8. Messenger RNA was enriched by Magnetic beads with Oligo (dT) according to the manufacturer's protocol and fragmented into short fragments. The mRNA products were used as templates to synthesize the first-strand cDNA using reverse transcriptase together with random hexamer primers. The second-strand cDNA was synthesized after adding reaction buffer, dNTPs, RNase H, DNA polymerase and MgCl_2_ with the remaining mRNA completely removed. Double stranded cDNA was purified using the QIAquick PCR Purification Kit according to the manufacturer’s protocol and end-repaired with T4 DNA polymerase and Klenow DNA polymerase. An “A” base was added to the 3’ end of DNA fragments. The cDNA was ligated to sequencing adapters with a ‘T’ base overhang at the 3’ end. DNA fragments with lengths from 250 bps to 500 bps were selected from electrophoresis gels and purified before PCR amplification of 15 cycles. The cDNA libraries were validated through the Agilent 2100 Bioanalyzer and ABI StepOnePlus Real-Time PCR system. A paired-end sequencing protocol was performed on the platform of HiSeq2000 (Illumina) [17].

#### Sequencing data quality control

In order to ensure valid biological conclusions, quality control (QC) and data filtering on the raw data were performed strictly prior to further processes and bioinformatics analysis. As an evaluation of raw data quality, we calculated the composition and distribution of bases (A, T, C, G, N) by using the FastQC program (version 0.11.2, http://www.bioinformatics.babraham.ac.uk/projects/fastqc/). The percentage of A/G should almost equal that of T/C and the rate of N should be less than one per cent. The proportion of low quality (Qs < 20) bases should be less than 10 per cent. If not, a re-sequencing process was activated. In the data filter process, dirty reads with sequencing adapters, reads with N rates greater than 10 per cent and those with more than 50 per cent low quality (Qs < 20) were discarded.

#### Alignment with reference sequences

In order to select a program for alignment, we compared SOAPaligner/Soap2 with other aligners including Bowtie, SOAP and MAQ. We found SOAPaligner/Soap2 performed better than others. SOAPaligner/Soap2 increased aligning speed dramatically and achieved better alignment rates for total and unique mapping [18]. Therefore, SOAPaligner/Soap2 (version 2.21) was chosen to align clean reads of each sample against the Morex genome (IBSC) [19] with a seed length of 32, allowing at most five mismatches in a read and maximal insert size of 1000 bp.

#### QC of alignments

SOAPcoverage was used for QC of the alignments. Coverage, depth and mapped rates (including unique mapped reads, total mapped read and unmapped reads) were assessed.

#### Differential expression analysis

The mRNA levels of genes were expressed by RPKM (Reads Per Kilo base-pair per Million mapped reads), which can eliminate the effects of gene length and data amount.

RPKM=106CNL103

Provided RPKM is the expression level of gene X, C is the number of reads mapped perfectly on gene X, N is the total number of reads uniquely mapped on the reference genes and L is the length of gene X. A strict algorithm—developed on the theoretical basis of the Poisson distribution and Multiple Testing [20]—was used to calculate FDR and screen DEGs between different groups [21]. The significance of differentially expressed genes under different conditions was judged by a stringent threshold of FDR ≤ 0.001 and an absolute value of log_2_ ratio ≥ 1.

#### Alternative splicing transcript identification

In order to detect alternative splicing evens, we had evaluated a few software for detecting splicing junctions, including TopHat, SpliceMap and MapSplice and SOAPsplice (version 1.10) [22]. We selected SOAPsplice for this work, since it produced the highest call rate (detected true junction number/true junction in total) and the lowest false positive rate (detected false junction number/the number of junctions all detected). Firstly, SOAPsplice mapped complete reads to the reference genome. Secondly, the unmapped (IUM) reads were mapped with the spliced alignment algorithm. Basically, SOAPsplice divided the IUM reads into two segments, which were derived from different exons in the premature mRNA. SOAPsplice first detected the longest 5' end segment of an IUM read that was mapped to the reference, then aligned the remaining segment to the reference sequences. Major criteria were:

1. Each segment should be longer than 8 bp.
2. No mismatch and no gaps were allowed in the alignment of each segment.
3. Distance of two segments was expected to range between 50 bp and 50,000 bp. Majority of known intron size was in this range in Eukaryote [23].
4. The boundary nucleotides of an intron should be in the form of "GT-AG", "GC-AG" or "AT-AC". When spliced alignment produced multiple hits, a splice junction candidate with "GT-AG" boundary was given the highest priority, followed by candidates with "GC-AG" and "AT-AC" boundaries [24].
5. When the segments had multiple hits to the reference, consideration was given to the cases where one segment had a unique hit while the other had multiple hits. The closest pair of hit was reported for this read. SOAPsplice ignored the other cases.
6. when the reads spanned more than 2 exons, an additional step was applied to detect junctions with reads longer than 50 bp. For the reads between 50 bp—100 bp, SOAPsplice spliced the read into two equal size segments. With reads longer than 100 bp, SOAPsplice spliced the reads into multiple segments of 50 bp from the 5' end until the remaining segment was in length between 50 and 100 bp, which was then spliced into two equal segments. SOAPsplice considered each segment as a sub-read, and treated it with the above alignment protocol.
7. Finally, SOAPsplice checked and concatenated the separated alignment hits for sub-reads to build the alignment for the original read [22].

#### Functional and pathway analysis

To investigate the biological implications, AS transcripts were mapped onto biological pathways using the blast tool in http://www.genome.jp/kaas-bin/kaas_main [25]. The AS transcripts were those with the same type of AS occurred in at least three out of four barley varieties at 24 h and 48 h. The protein sequences were downloaded from IBSC database (ftp://ftpmips.helmholtz-muenchen.de/plants/barley/public_data/) and used for the mapping.

The RNA-seq data have been submitted to NCBI GEO database (accession number GSE66024).

## Results

### RNA-seq read stat istics and alignments to barley reference genome

RNA-seq generated about 26 million reads which accounted for at least 2.3 G base pairs for each barley library (S1 Table). More than 67% and 45% of the reads were mapped to genomic and annotated gene sequences, respectively (International Barley Sequencing Consortium, IBSC) [19]. Between 7800 and 8700 novel transcripts were detected in the eight libraries (S2 Table), which were equivalent to about 30% of high confident genes. Gene coverage was used to calculate the percentage of a gene covered by the reads. The RNA-seq data showed that the majority of the genes (60%) had 90–100% gene coverage and another 25% of genes had at least 50% gene coverage (S3 Table).

### Numbers and types of AS events in germinating embryos

Large numbers of AS events (2635–3901) or genes (2229–3201) were detected in the germinating barley embryo (Table 1). About 1.2 AS events per AS gene were estimated (Table 1). The current gene annotation in barley has 15754 intron-containing genes [19]. Thus, 14–20% of intron-containing genes underwent AS during in germinating barley embryos. Moreover, alternative 3’ splicing (A3S) was the major AS event in the germinating barley embryo, accounting for 39–46% of total AS events, while intron retention (IR) and alternative 3’ splicing (A5S) accounted for 29–36% and 18–23% of total AS events, respectively. Of other AS event types, 2–3% was exon skipping, while alternative first exon and alternative last exon were negligible, and the mutually-exclusive exon was not detectable. When we calculated the numbers of AS transcripts at 24 h and 48 h germination, we have found that a half or over a half AS transcripts detected at 24 h had disappeared at 48 h (45–74%, Table 1). Almost same number of new AS transcripts were detected at 48 h germination. When analyses for common AS transcripts detected in at least three out of four barley varieties at 24 h and 48 h, the number of AS genes were 669 and 549 at 24 h and 48 h, respectively (Table 2). The percentages of A3S, IR and A5S transcripts over total AS transcripts were 34–45%, 32–34% and 16–21%, respectively. Once again, about a half of commonly detected AS transcripts identified at 24 h were not present at 48 h (Table 2).

**Table 1:** Alternative splicing events and genes.

Varieties	AS types	Events		Genes		Turnover
		24 h	48 h	24 h	48 h	
**Bass**	**Alternative 3' splicing**	1710 (43)	1203 (42)	1388	1037	888 (64)
	**Intron retention**	1166 (29)	1033 (36)	927	849	501 (54)
	**Alternative 5' splicing**	823 (21)	521 (18)	725	462	537 (74)
	**Exon skipping**	189 (4)	81 (2)	148	68	
	**Alternative first exon**	12 (0)	8 (0)	12	8	
	**Alternative last exon**	1 (0)	3 (0)	1	3	
	**Total**	**3901**	**2849**	**3201**	**2427**	
**Harrington**	**Alternative 3' splicing**	1217 (46)	1148 (43)	1022	979	603 (59)
	**Intron Retention**	781 (29)	836 (31)	635	737	286 (45)
	**Alternative 5' splicing**	547 (20)	559 (21)	490	511	319 (65)
	**Exon skipping**	74 (2)	87 (3)	66	72	
	**Alternative first exon**	14 (0)	10 (0)	14	10	
	**Alternative last exon**	2 (0)	1 (0)	2	1	
	**Total**	**2635**	**2641**	**2229**	**2310**	
**Stirling**	**Alternative 3' splicing**	1447 (40)	1180 (39)	1213	1004	716 (59)
	**Intron Retention**	1199 (33)	1046 (35)	965	833	521 (54)
	**Alternative 5' splicing**	746 (21)	627 (21)	649	565	422 (65)
	**Exon skipping**	130 (3)	92 (3)	101	77	
	**Alternative first exon**	12 (0)	3 (0)	12	3	
	**Alternative last exon**	3 (0)	3 (0)	3	3	
	**Total**	**3537**	**2951**	**2943**	**2485**	
**Baudin**	**Alternative 3' splicing**	1504 (43)	1287 (43)	1216	1083	717 (59)
	**Intron Retention**	1015 (29)	897 (30)	823	728	453 (55)
	**Alternative 5' splicing**	795 (23)	646 (21)	698	575	475 (68)
	**Exon skipping**	116 (3)	107 (3)	101	91	
	**Alternative first exon**	8 (0)	14 (0)	8	14	
	**Alternative last exon**	3 (0)	5 (0)	3	5	
	**Total**	**3441**	**2956**	**2849**	**2496**	

AS events were identified using software POAPsplice [22]. Numbers in brackets indicate % of individual AS events over total AS events. Zeroes indicate less than 0.5%. The mutually-exclusive exon type of AS was not detected in barley. The last column (turnover) shows number of AS transcripts detected at 24 h, but disappeared at 48 h, indicating a quick turnover phenomenon. The number in the brackets indicates % of transcripts disappeared at 48 h.

**Table 2:** Number of AS transcripts commonly occurring in at least three out of four barley varieties.

AS types	24 h	48 h	Turnover
**A3S**	299 (45)	228 (34)	155 (52)
**IR**	229 (34)	213 (32)	105 (46)
**A5S**	141 (21)	108 (16)	80 (57)
**Total**	669	549	

We have identified common AS transcripts occurring in at least three out of four barley varieties at 24 h and 48 h. Number in the brackets indicate the % of each type of AS transcripts over total AS genes. The last column (turnover) is the number of AS transcripts appeared at 24 h, but disappeared at 48 h. The numbers in brackets indicate % of AS transcripts disappeared at 48 h.

### Mapping AS transcripts onto biological pathways

The AS transcripts were mapped on to KEGG pathways of ribosome, RNA transport, spliceosome, oxidative phosphorylation, mRNA surveillance and over 100 other biological pathways (S4–S10 Tables). The top 20 pathways with most AS transcript numbers were mainly responsible for protein synthesis (ribosome, RNA transport, protein processing in endoplasmic reticulum and biosynthesis of amino acids), energy and carbon metabolism (glycolysis, starch and sugar metabolism and carbon metabolism) and post-transcriptional regulation (spliceosome, and ubiquitin mediated proteolysis). The genes undergone AS in germination embryos also included those coding for membrane repair, phytohormone responsive (S4–S10 Tables) and cell wall modification [6]. An auxin efflux carrier (MLOC_12686), three AUX/IAA responsive transcripts (MLOC_ *MLOC_54787*, *MLOC_75842* and *MLOC_65332*) and a serine/threonine protein kinase SRK2 (SnRK2) (*MLOC_22145*) gene were among them.

### Transcription of AS genes

The mRAN levels of AS genes were shown in S11–S16 Tables. Some of the AS genes could be highly expressed in the germinating embryos (>1000 RPKM). The mRNA levels were low to intermediate for most of the genes (10–1000 RPKM). There was not significant difference in transcript levels for most genes between 24 h and 48 h.

### Gene expression correlation analysis

**Fig 1:**
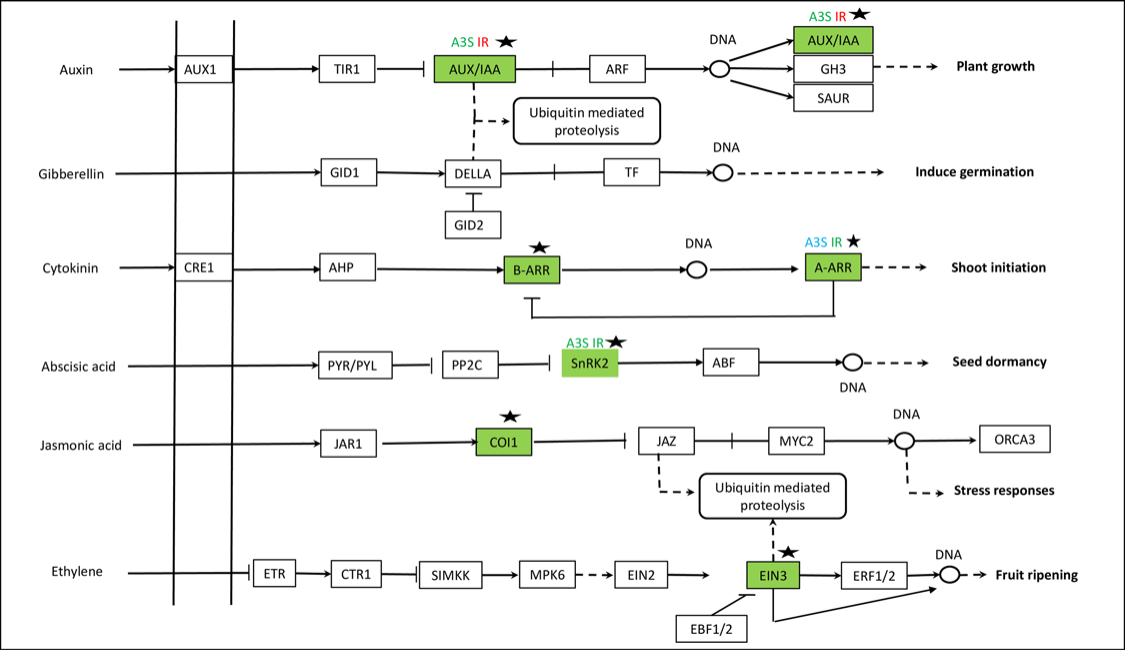
AS transcripts on plant hormone signal transduction pathway. AS transcripts were indicated by A3S, IR or A5S on tops of the corresponding genes. Blue and red colors indicated AS at 24 h and 48 h, respectively, while green color indicated that AS was detected at both 24 h and 48 h. AUX/IAA: Auxin-responsive protein IAA (MLOC_65332, A3S, MLOC_54787, IR and MLOC_75842, IR); JAZ: Jasmonate ZIM domain-containing protein (MLOC_12120, IR); ARR-A: Two-component response regulator A family (MLOC_64100, A3S, IR, MLOC_14492, A3S); SnRK2: Serine/threonine-protein kinase SRK2 (MLOC_69212,A3S, IR; MLOC_3013, IR; MLOC_63787, A3S, MLOC_22145, A3S, IR). The black stars indicated mRNA levels at 54–100 RPKM. AUX1: Auxin influx carrier; TIR1: Transport inhibitor response 1; AUX/IAA: Auxin-responsive protein IAA (MLOC_58812, MLOC_60624, MLOC_54255); ARF: Auxin response factor; GH3: Auxin response GH3 gene family; SAUR: SAUR family protein; GID1: Gibberellin receptor GID1; DELLA: DELLA protein; GID2: F-box protein GID2; TF: Phytochrome-interacting factor 4; CRE1:Cytokinin receptor; AHP: Histidine-containing phosphor-transfer protein; B-ARR: Two-component response regulator ARR-B (MLOC_3955); ARR-A: Two-component response regulator A family (MLOC_64100); PYR/PYL: Abscisic acid receptor PYR/PYL family; PP2C: Protein phosphatase 2C; SnRK2: Serine/threonine-protein kinase SRK2 (MLOC_62759, MLOC_69212); ABF: ABA response element binding factor; JAR1: Jasmonic acid-amino synthetase; COI1: Coronatine-insensitive protein1 (MLOC_4800); JAZ: Jasmonate ZIM domain-containing protein (MLOC_12120); MYC2: Transcription factor MYC2; ETR: Ethylene receptor; CTR1: Serine/threonine-protein kinase; SIMKK: Mitogen-activated protein kinase kinase; MPK6: Mitogen-activated protein kinase C; EIN2: Ethylene-insensitive protein 2; EIN3: Ethylene-insensive protein 3 (MLOC_14619); EBF1/2: EIN3-binding F-box protein; ERF1: Ethylene-responsive transcription factor 1.

Coordinative regulation of phytohormones play key roles in seed germination [26]. After identifications of several AS hormone responsive transcripts (Fig 1), we conducted gene expression correlation analyses to investigate co-expressed genes in germinating embryos. The co-expressed genes with high correlation values (> = 0.9 or < = -0.9) were then mapped to KEGG pathways (http://www.genome.jp/kaas-bin/kaas_main). These co-expressed genes were mapped onto 50 to 120 KEGG pathways (Table 3, S17–S21 Tables). Genes coding for Auxin efflux carrier and Auxin responsive proteins (AUX/IAA) had many co-expressed genes, while Serine/threonine protein kinase SRK2 gene (SnRK2) had fewer co-expressed genes in protein synthesis and energy metabolism pathways (Table 3).

**Table 3:** Co-expression of AS hormone responsive genes with genes responsible for protein synthesis and energy metabolism.

Pathways	PIN *MLOC_12686*	AUX/IAA *MLOC_54787*	AUX/IAA *MLOC_75842*	SnRK2 *MLOC_22145*
**Ribosome**	5	2	12	0
**Biosyn AA**	8	3	2	2
**RNA transport**	9	7	1	0
**Carbon Met**	9	6	3	2
**Oxidative phos**	4	2	0	0
**Spliceosome**	1	6	0	0
**Plant h s trans**	1	2	1	3

Co-expression of AS auxin efflux carrier (PIN), auxin responsive protein (AUX/IAA) and serine/threonine protein kinase SRK2 (SnRK2) genes with other genes were analysed. The co-expressed genes with high correlation values (> = 0.9 or < = -0.9) were mapped onto KEGG pathways (http://www.genome.jp/kaas-bin/kaas_main). The number of co-expressed genes for each hormone responsive protein were listed for ribosome, biosynthesis of amino acid (Biosy AA), RNA transport, carbon metabolism (Carbon Met), oxidative phosphorylation (Oxidative phos), spliceosome and plant hormone signal transduction (Plant h s trans) pathways.

#### Auxin efflux carrier

Auxin efflux carrier transcript (*MLOC_12686*) underwent IR type of AS (S10 Table). It had over 354 co-expressed genes (S17 Table). They were mapped onto over 280 KEGG pathways (S17 Table). Pathways of protein synthesis (ribosome, biosynthesis of amino acid, RNA transport) and energy metabolism (carbon metabolism, oxidative phosphorylation) were on the top of the list with most gene numbers. It also co-expressed with many genes functioning in DNA replication, cell cycle, purine metabolism and nucleotide excision repair pathways. Expression of auxin efflux carrier gene was negatively correlated with expressions of several metabolic genes in glycolysis and TCA cycle (Table 4).

**Table 4:** Co-expression of AS hormone responsive genes with genes functioning in energy metabolism pathways.

AS hormone responsive proteins	Co-expressed genes	Corr values
Auxin efflux carrier	Hexokinase (MLOC_53317)	-0.93
(MLOC_12686)	Glyceraldehyde-3-phosphate dehydrosenase (MLOC_18233)	-9.97
	Pyruvate dehydrogenase E1 (MLOC_53947)	0.92
	Malate dehydrogenase (MLOC_61949)	-0.92
	Citrate synthase (MLOC_66594)	-0.92
AUX/IAA	Hexokinase (MLOC_53317)	0.94
(MLOC_54787)	Glucose-6-phosphate isomerase (MLOC_1497)	0.92
	6-Phosphofructokinase (MLOC_67234)	0.94
	6-Phosphofructokinase (MLOC_11145)	0.93
	Pyruvate dehydrogenase E1 (MLOC_63372)	0.93
	Isocitrate dehydrogenase (MLOC_69600)	0.9
AUX/IAA (MLOC_75842)	Glyceraldehyde-3-phosphate dehydrosenase (MLOC_52515)	0.94
SnRK2 (MLOC_22145)	2,3-Bisphophoglycerate-independent phosphoglycerate mutase (MLOC_52687)	-0.96

Co-expression of AS auxin efflux carrier, auxin responsive protein (AUX/IAA) and serine/threonine protein kinase SRK2 (SnRK2) genes with energy metabolism genes were analysed. The co-expressed genes with high correlation values (> = 0.9 or < = -0.9) were listed. Corr: correlation

#### AUX/IAA

We identified three AS *AUX/IAA* transcripts (*MLOC_54787*, *MLOC_75842* and *MLOC_65332*). Two of them (*MLOC_54787* and *MLOC_75842*) underwent IR type of AS (S10 Table), while the third (*MLOC_65332*) underwent A3S (S5 and S6 Tables). Both IR *AUX/IAA* transcripts have over 320 co-expressed genes (S18 and S19 Tables). These co-expressed genes were mapped onto over 100 KEGG pathways. The genes co-expressed with AS *AUX/IAA* gene (*MLOC_75842*) was preferably mapped onto ribosome pathway, but the genes co-expressed with other *AUX/IAA* gene *(MLOC_54787)* were preferably mapped on to carbon metabolism and oxidative phosphorylation pathways (Table 3). The AS *AUX/IAA* gene (*MLOC_54787*) could play more important roles in energy metabolism, while the other (*MLOC_75842*) was likely related to protein synthesis as shown by the number of co-expressed genes in corresponding pathways (Table 3). Unlike AS auxin efflux carrier, expressions of both IR *AUX/IAA* genes were positively correlated with expressions of several genes in glycolysis and TCA cycle (Table 4). The third A3S *AUX/IAA* gene (*MLOC_65332*) had only nine co-expressed genes (S21 Table).

#### SnRK2

*SnRK2* gene (SnRK2, *MLOC_22145*) underwent both A3S and IR type of AS (Fig 1, S6 Table, S10 Table). It had 138 co-expressed genes (S20 Table). Two co-expressed genes were mapped to carbon metabolism pathways and three co-expressed genes were mapped onto spliceosome. It was negatively correlated with the expression of a gene in glycolysis (*MLOC_52687*, Table 4).

## Discussion

### Number of AS transcripts in germinating embryos

We have identified a large number of AS transcripts in geminating embryos (Tables 1 and 2). The total number of AS transcripts is roughly consistent at a certain time point in each barley variety (2200–3900). However, because AS is dynamically regulated and half turnover time is approximately 24 hours, i. e. about a half of the AS transcripts detected at 24 h have disappeared at 48 h and new AS transcripts are detected (Tables 1 and 2), number of genes undergoing AS could be very large during seed germination. Furthermore, the major AS type in barley embryos is the same as in various maize tissues [27], but differs from other plant species. In germinating barley embryos, the major AS type is A3S (34–45%), while in rice, *Sorghum bicolor*, maize, soybean and *Arabidopsis*, IR is the major AS type [28,29,30,31].

### Potential roles of AS in seed germination

AS regulates many different biological processes. It plays key roles in abiotic stress tolerance in plants[11]. Two AS variants of heat shock transcription factors are generated under heat stress in *Arabidopsis*—one targets for nonsense-mediated decay while the other short form binds to heat shock transcription factor promoter and activates self-transcription. Other stress-induced AS transcripts code for rice drought-regulated dehydration-responsive element binding protein 2 [32], potato cold-regulated invertase [33], invertase inhibitor [34] and indeterminate domain 14 transcription factor [35]. All AS variants of these transcripts have different biological functions and add additional capacities for environmental adaptations. Several splicing regulators also undergo AS in stress conditions including splicing regulators 30 and 45a in *Arabidopsis* [9]. Variants of AS transcripts also regulate dormancy and germination in plants. Transcripts of Viviparous 1 (VP1) [36] and ABI3 [37], have been detected to undergo AS. VP1 is a regulator of seed maturation. Mutation of VP1 gene disrupted seed maturation and promoted germination in maize [38,39]. Orthologues of maize VP1 genes were *ABI3* in *Arabidopsis* [40], *PtABI3* in poplar [41], *vp-1* in wheat [36], *Osvp1* in rice [42] and *Afvp1* in wild oat [43]. All variants of AS *VP1* orthologue transcripts had different effects on seed germination. For example, several variants of AS *VP1* transcripts were detected in wheat embryos [36]. Some of them causes pre-harvest sprouting [36]. In this study, we have identified AS transcripts commonly detected in four barley varieties. These AS transcripts could possibly affect protein synthesis, energy metabolism and post-transcriptional regulations, since they participated in the activities of these pathways as shown that they were predominately mapped on to ribosome, RNA transport, biosynthesis of amino acid, protein processing in endoplasmic reticulum, glycolysis, oxidative phosphorylation, spliceosome, ubiquitin-mediated proteolysis and plant signal transduction pathways (S4 Table).

Since VP1 gene played very important roles in both seed maturation and seed germination, we have closely examined the changes in its transcript level and AS patterns. Barley genome contains one annotated *VP1* gene (MLOC_69727) (IBSC, [19]). The transcript levels were consistent among the four barley varieties ranging at 20–32 RPKM (S11 Table). However, this gene underwent A3S at 24 h and 48 h, A5S at 48 h and IR type of AS at 48 h (S5 and S6 Tables, S8 and S10 Tables), implicating that AS variants of VP1 could be involved in seed germination.

Furthermore, synthesis of auxin was detected as early as the second day in germinating common bean seeds [44]. Auxin also plays key roles in control seed dormancy in a coordinative network of auxin and ABA signalling [45]. Gene expression correlation analysis showed that AS auxin efflux carrier and AUX/IAA transcripts could play key roles in regulating biological processes in germinating embryos, since expressions of these hormone responsive genes was correlated with the expression of genes in protein synthesis and energy metabolism pathways (Fig 1, Tables 3 and 4). Barley genome contained 12 annotated Auxin efflux carrier genes in chromosome 1H, 3H, 5H, 6H and 7H (IBSC). Only one of them from chromosome 7H (MLOC_12686) underwent IR type of AS at both 24 h and 48 h germination. This gene was expressed in all four barley varieties with mRNA levels ranging from 50–158 RPKM. Furthermore, this gene was co-expressed with 355 other genes. Many of them functioned in protein synthesis and energy metabolism (S17 Table).

## Conclusions

Alternative splicing could play important roles in seed germination. It was involved in most active biological processes including protein synthesis, carbon and energy metabolism in the germinating embryos.

## Supporting Information

S1 Table RNA-seq read statistics. Alignment of RNA-seq transcript to barley genomic DNA and annotated genes.
(XLSX)

S2 Table Novel transcripts detected in 4 barley varieties. (XLSX)

S3 Table Gene coverage statistics-the percentage of a gene covered by reads. (XLSX)

S4 Table Number of AS transcripts mapped onto KEGG pathways using the blasting tools. (XLSX)

S5 Table A3S transcripts detected in at least three out of four varieties (Bass, Harrington, Stirling and baudin) at 24 h in germinating embryos. (XLSX)

S6 Table A3S transcripts detected in at least three out of four varieties (Bass, Harrington, Stirling and baudin) at 48 h in germinating embryos. (XLSX)

S7 Table A5S transcripts detected in at least three out of four varieties (Bass, Harrington, Stirling and baudin) at 24 h in germinating embryos. (XLSX)

S8 Table A5S transcripts detected in at least three out of four varieties (Bass, Harrington, Stirling and baudin) at 48 h in germinating embryos. (XLSX)

S9 Table IR type of AS transcripts detected in at least three out of four varieties (Bass, Harrington, Stirling and baudin) at 24 h in germinating embryos. (XLSX)

S10 Table IR type of AS transcripts detected in at least three out of four varieties (Bass, Harrington, Stirling and baudin) at 48 h in germinating embryos. (XLSX)

S11 Table The mRNA levels of commonly detected A3S transcripts at 24 h. (XLSX)

S12 Table The mRNA levels ofcommonly detected A3S transcripts at 48 h. (XLSX)

S13 Table The mRNA levels of commonly detected A5S transcripts at 24 h. (XLSX)

S14 Table The mRNA levels of commonly detected A5S transcripts at 48 h. (XLSX)

S15 Table The mRNA levels of commonly detected IR transcripts at 24 h. (XLSX)

S16 Table The mRNA levels of commonly detected IR transcripts at 48 h. (XLSX)

S17 Table Co-expressed genes with AS auxin efflux carrier gene (MLOC_12686). (XLSX)

S18 Table Co-expressed genes with AS auxin responsive gene (MLOC_54787). (XLSX)

S19 Table Co-expressed genes with AS auxin responsive gene (MLOC_75842). (XLSX)

S20 Table Co-expressed genes with AS SRK2 (MLOC_22145). (XLSX)

S21 Table Co-expression analysis of AS Auxin responsive protein (MLOC_65332). (XLSX)

## Abbreviations

ABI3: abscisic acid insensitive 3
ABI5: abscisic acid insensitive 5
AS: alternative splicing
AUX1: auxin efflux carrier
AUX/IAA: Auxin responsive protein
DEG: differentially expressed genes
JAZ: Jasmonate ZIM domain-containing gene
RPKM: reads per kilo base transcriptome per million mapped reads
SnRK2: Serine/threonine protein kinase SRK2 gene
VP1: viviparous 1
